# Impact of Nutritional Status on Pulmonary Function in Pediatric Cystic Fibrosis: A Retrospective Multicenter Study from Upper Egypt

**DOI:** 10.3390/medsci13030165

**Published:** 2025-09-01

**Authors:** Khaled Saad, Eman F. Gad, Samaher F. Taha, Sherin A. Taha, Hamada K. Fayed, Mahmoud Elsaeed, Thamer A. M. Alruwaili, Mohamed Fahmy M. Ibrahim, Amira Elhoufey, Ahmed M. Esmat Mansour, Amir M. Aboelgheet

**Affiliations:** 1 Department of Pediatrics, Faculty of Medicine, Assiut University, Assiut 71515, Egypt; 2 Department of Pediatrics, Faculty of Medicine, Suez University, Suez 43221, Egypt; 3 Department of Chest Diseases, Faculty of Medicine, Al-Azhar University, Assiut 71111, Egypt; 4 Department of Chest Diseases, Faculty of Medicine, Al-Azhar University, Cairo 11884, Egypt; 5 Department of Pediatrics, College of Medicine, Jouf University, Sakaka 72388, Saudi Arabia; 6 Department of Pediatrics, Faculty of Medicine, Al-Azhar University, Assiut 71111, Egypt; 7 Department of Community Health Nursing, Faculty of Nursing, Assiut University, Assiut 71515, Egypt; 8 Department of Community Health Nursing, Alddrab University College, Jazan University, Jazan 45142, Saudi Arabia; 9 Department of Chest Diseases, Faculty of Medicine, Menoufia University, Menoufia 32511, Egypt

**Keywords:** cystic fibrosis, pediatrics, malnutrition, pulmonary function

## Abstract

**Aim:** This study aimed to evaluate the nutritional status of children with cystic fibrosis (CF) and investigate the correlation between malnutrition and the decline of pulmonary function in this population. **Methods:** We retrospectively analyzed the clinical data of children with CF admitted to four large tertiary centers in Upper Egypt. We compared clinical characteristics among children with different nutritional statuses and evaluated the correlation between malnutrition and pulmonary functions. **Results:** A total of 104 children with CF, including 54 males (52%), aged 3 to 18 years, were analyzed. Respiratory symptoms were present in all cases (100%). Malnutrition was observed in 72% (75/104) of the participants, with affected children exhibiting significantly lower body weight and serum albumin levels. Pulmonary function tests showed that vital capacity (VC) and the predicted values for forced vital capacity (FVC), forced expiratory volume in 1 s (FEV_1_), FEV_1_/FVC, and expiratory flow at 25%, 50%, and 75% of FVC were all lower in the malnourished group compared to children with normal nutrition. Correlation analysis demonstrated that the body mass index (BMI) Z-score was positively correlated with these pulmonary function indicators. **Conclusions:** Malnutrition is highly prevalent among Egyptian children with CF and is associated with decreased pulmonary function. Improving nutritional status may enhance lung function in this population.

## 1. Introduction

Cystic fibrosis (CF) is a severe and progressive, inherited, multisystem disorder primarily caused by mutations in the cystic fibrosis transmembrane conductance regulator (CFTR) gene. In children, CF manifests as progressive obstructive lung disease, chronic sinusitis, pancreatic insufficiency, malnutrition, CF-related liver disease, and, in some cases, diabetes mellitus. CF is one of the most common inherited autosomal recessive disorders among populations of European descent, with an incidence of 1 in 2500 to 3500 live births [1,2]. However, the prevalence varies significantly across regions. CF affects approximately 100,000 individuals worldwide, with about 40,000 patients in the US alone [2]. In contrast, CF is considered rare in Asian populations, with reported incidences below 1 in 100,000 live births [3]. In the Middle East and North Africa (MENA) region, including Egypt, the true burden of CF remains underrecognized due to limited newborn screening programs, diagnostic challenges, and underreporting. However, due to high rates of consanguinity—reported in up to 40% of marriages in some countries—the prevalence of autosomal recessive disorders like CF is likely underestimated. Recent studies suggest a rising number of diagnosed cases in the MENA region, with Egypt reporting an increasing number of CF cases, though precise epidemiological data are still lacking [3,4]. Over the past few decades, advances in diagnosis and management, including newborn screening, improved pulmonary care, and lung transplantation for end-stage disease, have dramatically improved survival for individuals with CF [1,2]. However, few studies on CF in Egyptian children exist, most of which are limited by small sample sizes and primarily focus on respiratory management and imaging features [3]. Nutritional issues, despite their critical role in disease progression and prognosis, have received relatively little attention in this population. Malnutrition and growth disorders are common among children with CF and are strongly linked with raised morbidity, mortality, and impaired quality of life [2,3]. Given the scarcity of data on CF in Egyptian children, our retrospective multicenter study analyzes clinical data focusing on nutritional status, clinical characteristics, and pulmonary functions. The primary aim of this study is to evaluate the nutritional status of children diagnosed with CF in Upper Egypt. The secondary objective is to investigate the association between malnutrition and pulmonary function impairments, as assessed by key pulmonary function parameters. This study aims to deepen clinicians’ understanding of the crucial role of nutritional management in CF, with the goal of improving lung function, growth, development, and overall prognosis in affected pediatric patients.

## 2. Patients and Methods

### 2.1. Patients

A total of 104 children diagnosed with CF at four tertiary centers in Assiut, Egypt, from July 2014 to June 2024 were selected for this study. CF was diagnosed according to previously published criteria [4]. We excluded patients with an unconfirmed diagnosis of CF, having other coexisting diseases affecting growth, or missing data in their files.

### 2.2. Methods

#### 2.2.1. Data Collection

We collected clinical data from children diagnosed with CF at four tertiary centers in Assiut, Egypt, between July 2014 and June 2024. The collected data included demographic information (sex and age) and anthropometric measurements (weight, height, and body mass index [BMI]). All anthropometric measurements were plotted on age-specific percentile curves, and Z-scores were calculated using WHO AnthroPlus software, version 3.2, in accordance with standardized growth assessment protocols. Additional data encompassed medical and family history, physical examination findings, and clinical manifestations. Laboratory test results, genetic testing outcomes, and sweat test results were also systematically recorded.

Standard spirometry was performed on 79 patients using a fully equipped computerized system (Cosmed SrL, Quark PFTs, ergo, Rome, Italy). The testing technique adhered to the official guidelines set by the American Thoracic Society recommendation for the standardization of spirometry [4]. The pulmonary function tests were repeated three times, with adequate rest provided between each trial. The best result of the three reproducible tests was taken as the final measurement for each individual. The following parameters were measured during the tests: vital capacity (VC), forced vital capacity (FVC), forced expiratory volume in 1 s (FEV_1_), FEV_1_/FVC ratio, peak expiratory flow rate (PEF), and forced expiratory flow (FEF) at 25%, 50%, 75% of forced vital capacity as a percentage of the predicted value.

Nutritional risk was assessed using the Screening Tool for the Assessment of Malnutrition in Pediatrics (STAMP). STAMP was selected for its validated effectiveness in pediatric populations and ease of use in clinical settings [5]. STAMP comprises three domains: (i) underlying medical condition, (ii) nutritional intake (including reduced oral intake or reliance on nutritional supplements), and (iii) anthropometric measurements (weight and height, compared to age- and sex-matched reference data). Each domain is scored based on predefined clinical criteria: the medical condition is scored from 0 to 2 (e.g., 0 = no chronic illness, 1 = chronic illness without complications, and 2 = chronic illness with complications); nutritional intake is scored from 0 to 1 (0 = normal intake and 1 = reduced intake or use of supplements); and anthropometric status is scored from 0 to 2 (0 = normal BMI/weight-for-height, 1 = mild deviation, and 2 = moderate to severe deviation). The scores from these three domains are summed up to estimate the overall risk of malnutrition, which is categorized into 3 levels: no/low risk (scores 0 to 1), moderate risk (scores 2 to 3), and high risk (scores ≥ 4).

Nutritional status was evaluated according to the 2006 World Health Organization Child and Adolescent Growth Standards [6]. We calculated the BMI Z-score for age using the WHO Anthro software for children aged 0–5 years and the WHO AnthroPlus software for those older than 5 years. Malnutrition severity was classified based on BMI Z-scores: Mild malnutrition: Z-score between >−2 and ≤−1. Moderate malnutrition: Z-score between >−3 and ≤−2. Severe malnutrition: Z-score ≤ −3 [7]. The prevalence of malnutrition was determined by dividing the number of children with mild, moderate, or severe malnutrition by the total number of cases.

#### 2.2.2. Statistical Analysis

Statistical analysis was carried out using IBM SPSS Statistics version 27.0. Continuous variables were evaluated for normality using the Shapiro–Wilk test, assessment of skewness and kurtosis, and visual inspection of histograms and Q–Q plots. Those following a normal distribution were summarized as mean [standard deviation (SD)] and compared across groups with the independent samples *t*-test. Variables deviating from normality were described using median values and interquartile ranges, and group comparisons were made using the Mann–Whitney U test. Categorical data were expressed as counts and percentages, and differences between groups were analyzed using the appropriate Chi-square test or Fisher’s exact test. To evaluate associations between variables, Pearson’s correlation coefficient was used for parametric data, while Spearman’s rank correlation coefficient was applied for non-parametric data. A multivariate linear regression model was constructed to determine the influence of selected clinical factors—specifically, age at evaluation, age at diagnosis of cystic fibrosis (CF), and BMI Z-score—on forced expiratory volume in one second (FEV_1_). Statistical significance was defined as a *p*-value < 0.05.

## 3. Results

One hundred four children with CF were included in our study, comprising 54 males (52%) and 50 females (48%), aged 3 to 18 years, with a mean age (SD) of 7.5 (3.3) years. Sweat chloride levels varied from 61 to 179 mmol/L, with a mean (SD) of 99 (36) mmol/L. Notably, 40% of participants reported consanguinity, while 7.6% had a family history of CF. The follow-up period for disease monitoring ranged from 1 to 12 years, with variability in duration based on individual patient presentation and clinical progression. Table 1 shows the clinical manifestations of all participants.

All patients included in this study were maintained on standard oral feeding. In cases where dietary intake was insufficient, oral nutritional supplements were administered to address caloric and nutrient deficits. Nutritional interventions were individualized based on the severity of malnutrition. Enteral feeding—via nasogastric or gastrostomy tubes—was initiated for patients presenting with severe malnutrition to ensure adequate energy intake and promote weight gain. Additionally, pancreatic enzyme replacement therapy (PERT) was systematically administered to all patients diagnosed with pancreatic insufficiency in alignment with established CF management guidelines. This intervention was implemented to optimize nutrient absorption and enhance growth outcomes. Table 1 shows the results of STAMP nutritional risk screening and BMI Z-scores. Table 2 compares the malnutrition groups (mild, moderate, and severe) and the normal nutrition group. Among the 104 pediatric patients included in this study, none (0%) were receiving highly effective CFTR modulator therapy, including elexacaftor/tezacaftor/ivacaftor (ETI). This is because CFTR modulators are not currently approved, funded, or available for clinical use in Egypt.

Seventy-nine pediatric patients with CF over 6 years of age completed pulmonary function tests, and 55 cases (77%) had abnormal pulmonary functions (Table 3). The malnutrition group showed significantly lower pulmonary function than the normal nutrition group. Table 3 presents the details of pulmonary function parameters for both the normal nutrition group and the three malnutrition subgroups. The correlation analysis between BMI Z-score and lung function parameters showed that the BMI Z-scores of children with CF have significant positive correlations with FEV 1% pred, FEV 1/FVC, FEF 25% pred, FEF 50% pred, and FEF 75% pred (Table 4). A multivariate regression analysis was conducted to determine whether patient clinical features influence FEV1. The variables included current age, age at CF diagnosis, BMI Z-score, and sex. The regression model revealed a significant negative association between current age and FEV1 (t = −2.093; *p* = 0.042). The BMI Z-score showed a significant positive association with FEV1 (t = 2.093; *p* = 0.037). No significant association was found between sex (t = 0.45; *p* = 0.60) or age at CF diagnosis (t = 1.63; *p* = 0.09) and FEV1.

## 4. Discussion

Individuals with CF experience a greater rate of malnutrition compared to the general population due to the disease’s impact on various organ systems, especially the pancreas [1,2,8]. The BMI Z-score and weight-for-length (WFL) Z-score are commonly used indicators to assess nutritional status in pediatric patients with CF. Studies suggest that the BMI Z-score may be more appropriate than the WFL Z-score, particularly for our patient group, as all are over 2 years old [9]. In our study, we utilized the BMI Z-score and found that the prevalence of malnutrition among children with CF was 72%. These findings emphasize the tremendous impact of malnutrition in pediatric patients with CF. Malnutrition remains a serious concern for patients with CF, particularly in resource-limited settings such as Egypt [3,10,11].

The high prevalence of malnutrition in our cohort aligns with findings from previous studies [10,11,12,13,14], which reported malnutrition frequencies from 45 to 89% in children with CF. Other studies found lower frequencies of malnutrition (10–30%) [15,16,17]. The causes of malnutrition in CF are multifactorial, including pancreatic insufficiency, the destruction of acinar pancreatic cells, the blockage of pancreatic ducts, and reduced enzyme activity, all contributing to malabsorption. This results in an inability to release bicarbonate and pancreatic enzymes into the duodenum, leading to a lack of macronutrient breakdown in the upper intestinal lumen and reduced neutralization of gastric contents [3,10].

Additionally, children with CF often have a reduced appetite, resulting in lower calorie intake, which worsens malnutrition [10,11,12]. Moreover, the energy requirements for people with CF are higher, estimated at 120–150% of normal needs. Infants and young children with CF are particularly vulnerable to severe malnutrition, which can cause stunted growth, poor cognitive function, and increased mortality [10,11,12]. Our study confirmed that malnutrition is highly prevalent among children with CF in Egypt. Some studies have suggested that the risk of malnutrition in children with CF increases with age [18,19].

The impact of CF on infant mortality and malnutrition in Egypt has not been investigated [3]. Malnutrition is a significant concern for individuals with CF, especially in our locality. Socioeconomic factors significantly contribute to malnutrition in people with CF in Egypt. Key barriers such as limited access to specialized CF care, financial constraints, and insufficient awareness regarding the critical role of nutritional interventions delay optimal CF management in low- and middle-income regions. Our study population included pediatric patients from four tertiary care centers, potentially representing a subset with comparatively better healthcare access than those in remote areas. However, the observed high malnutrition prevalence highlights the urgent need for public health initiatives to enhance CF care universally. Governmental and non-governmental organizations can play pivotal roles in addressing these challenges by ensuring the availability of essential medications, including pancreatic enzyme replacements and nutritional supplements, as well as by providing financial assistance to families impacted by CF. Additionally, health education campaigns may raise awareness about the importance of nutrition in CF management, promoting timely intervention-seeking behaviors among families.

The correlation between BMI Z-scores and pulmonary function tests in children with CF highlights the crucial role that nutritional status plays in respiratory health. Our findings indicate that higher BMI Z-scores are significantly associated with improved pulmonary function outcomes, including FVC, FEV_1_, the FEV_1_/FVC ratio, and PEF. Specifically, a positive correlation was noted between BMI Z-scores and these parameters, with the strongest correlation seen in the FEV_1_/FVC ratio, indicating that children with better nutritional status have greater lung volumes and better airflow efficiency. Our findings and previous studies [13,20,21] confirmed the positive correlations between nutritional status and pulmonary functions in patients with CF.

Lower BMI Z-scores, indicative of malnutrition, were associated with compromised pulmonary functions, which may be due to decreased muscle strength, including respiratory muscles, decreased muscle mass, and subsequent reduction in strength, impairing diaphragm performance and contributing to diminished respiratory capacity and recurrent lung infections, which further degrade pulmonary function and contribute to the overall poor health status that limits the child’s ability to maintain adequate pulmonary function [13,20].

The interplay between nutritional status and pulmonary function in CF is bidirectional. Repeated lung infections and declining lung function can exacerbate malnutrition, while malnutrition can further impair lung health. Active nutritional intervention has been shown to reduce the frequency of lung infections and improve pulmonary function. Kilinc et al. [13] reported that in patients with CF whose BMI Z-score improved during follow-up, the mean FEV_1_ increased by 14% from the baseline results. Steinkamp and Wiedemann [20] found that malnourished adolescents with CF aged 12–18 experienced a significant decline in FEV_1_ (about 20%), while those with normal weight maintained stable FEV_1_ levels above 80%. Moreover, malnourished patients across all age groups, as well as those infected with *P. aeruginosa*, had notably poorer lung functions and a greater annual reduction in predicted FEV_1_ compared to well-nourished individuals. Over one year, adolescents who lost more than 5% of their predicted weight have experienced an average FEV_1_ decrease of 16.5%, whereas those who gained weight showed an increase of 2.1% in predicted FEV_1_ [20].

Previous studies found that weight gain among children with CF correlated with greater FEV_1_ increases compared to those who lost weight. Moreover, improved nutritional status was linked to enhanced respiratory function. Furthermore, nutritional status in childhood may influence the severity of pulmonary dysfunction later in life [20,21,22]. A previous study [22] observed that a 10% increase in BMI in underweight children led to a 4% rise in FEV_1_, while patients with a normal BMI experienced a 5% increase in FEV_1_. These findings underscore the importance of early nutritional interventions to support optimal growth and development, potentially mitigating the decline in lung function in patients with CF. Interventions aimed at improving BMI could play a pivotal role in enhancing respiratory outcomes, reinforcing that nutritional management should be an integral part of the comprehensive care for children with CF. Previous studies showed that enteral nutrition significantly improved BMI Z-scores and reduced the decline in lung function among patients with CF [3,23]. Therefore, active treatment to maintain good nutrition and protect lung function is essential for children with CF. Nutritional treatment should be multidisciplinary, including dietary supplements, PERT, and possibly enteral feeding in cases of severe disease [3]. PERT is essential for patients with pancreatic insufficiency, which affects up to 85% of our cohort with CF, optimizing the digestion and absorption of nutrients, supporting weight gain, and improving overall nutritional status [24,25]. In addition to PERT, supplementation with fat-soluble vitamins (A, D, E, and K) is a cornerstone of care due to malabsorption caused by steatorrhea [24,25]. Deficiencies in these vitamins are common and can lead to significant complications: vitamin A deficiency may impair immune and visual function, vitamin D deficiency is associated with reduced bone mineral density and increased infection risk, vitamin E deficiency can result in neurological sequelae, and vitamin K deficiency may contribute to coagulopathy [12,24]. Therefore, routine monitoring of serum levels and administration of high-dose oral supplements are critical components of nutritional management in CF [12,25]. To meet the elevated energy requirements—typically 110–150% of the recommended daily allowance—high-calorie nutritional supplement drinks and ready-to-use therapeutic foods (e.g., lipid-based nutrient supplements) are frequently prescribed, particularly during periods of poor oral intake or acute pulmonary exacerbations [3,10]. Traditional nutritional guidelines for CF have historically recommended a high-fat diet to help address malabsorption and promote adequate weight gain [24]. However, more recent research indicates that high-fat intake should be individualized to each patient. Some studies have found that a very high fat intake may not be suitable for all patients with cystic fibrosis—especially for those who retain some pancreatic function or are taking highly effective CFTR modulator medications—as it can cause gastrointestinal discomfort or result in weight gain that exceeds what is considered healthy [26]. Personalized dietary planning based on fat absorption capacity, growth trajectory, and comorbidities such as CF-related liver disease or dyslipidemia is now recommended [26].

Emerging research highlights the role of the gut microbiome in CF pathophysiology. Intestinal dysbiosis—characterized by reduced microbial diversity, the overgrowth of pathogenic bacteria (e.g., *Escherichia coli* and *Staphylococcus*), and the depletion of beneficial taxa such as *Bifidobacterium* and *Lactobacillus*—is frequently observed, even in early life. This dysbiosis contributes to chronic intestinal inflammation and impaired nutrient absorption, and may influence pulmonary outcomes via the gut–lung axis [27,28]. In this context, probiotics and prebiotics have emerged as potential adjunctive therapies. Specific probiotic strains, including *Lactobacillus rhamnosus GG* and *Bifidobacterium lactis*, have demonstrated benefits in improving gastrointestinal symptoms and possibly reducing the frequency of pulmonary exacerbations [27,28]. A Cochrane review reported moderate-certainty evidence for the use of probiotics in reducing pulmonary exacerbations in children with CF. However, long-term safety and strain-specific efficacy require further investigation [28]. Prebiotics may promote the growth of beneficial bacteria and enhance short-chain fatty acid production, supporting gut barrier integrity. However, responses to microbiome-targeted interventions vary widely, underscoring the need for individualized approaches based on baseline microbiota, dietary patterns, and medication use [29].

The advent of highly effective CFTR modulators—such as ETI—has significantly altered the nutritional landscape of CF. These therapies correct the underlying CFTR protein defect, leading to rapid improvements in weight, BMI, and fat absorption, often independent of increased caloric intake [30,31]. However, in our cohort, none were receiving ETI or any other CFTR modulator therapy, as these medications are not yet approved in Egypt due to regulatory and financial barriers. Studies have shown that treatment with ETI is associated with increased fecal elastase levels and improved serum concentrations of fat-soluble vitamins, indicating enhanced intestinal absorptive capacity [30,31,32,33]. However, this therapeutic success brings new challenges: overweight and obesity are increasingly reported among people with CF (PwCF). Data from a cross-sectional analysis of patients with CF revealed that 25% of those with severe CFTR genotypes (typically associated with pancreatic insufficiency) had a BMI ≥ 25 kg/m^2^ between 2015 and 2017 [6,32]. This shift necessitates a reevaluation of nutritional strategies—from a historical focus on preventing undernutrition to avoid excessive weight gain and associated metabolic complications, including CF-related diabetes (CFRD) and metabolic dysfunction-associated steatotic liver disease (MASLD) [6,32]. The 2024 European Guidelines on Nutrition Care for CF recommend maintaining BMI above the 50th percentile in children but also caution against uncontrolled weight gain, particularly in the modulator era [33].

### 4.1. Limitations of the Study

The retrospective nature of this study limits our ability to establish causal relationships between malnutrition and pulmonary outcomes. Since participants were recruited from tertiary centers, they likely represent patients with better access to specialized CF care. This may introduce a selection bias and lead to an underestimation of the severity of malnutrition and poorer outcomes among children in less advantaged settings. Additionally, the study did not include detailed data on factors affecting FEV1, such as colonization and other management approaches, which were not included in the analysis and may influence the results. While changes in BMI Z-score and lung function were observed, the study lacked detailed longitudinal follow-up data to evaluate how sustained improvements in nutrition affect pulmonary function over time. Moreover, while malnutrition was identified, there was limited information on the specific nutritional interventions used and their effectiveness in improving patient outcomes.

### 4.2. Future Research Directions

While our study provides valuable insights into the relationship between nutritional status and pulmonary function in children with CF, several areas warrant further research. Longitudinal studies are needed to explore the long-term effects of early nutritional interventions on pulmonary outcomes and overall survival in patients with CF. Additionally, research into developing novel nutritional supplements and therapeutic strategies that address the specific needs of patients with CF could further improve outcomes.

## 5. Conclusions

Our study demonstrates that malnutrition is highly prevalent among children with CF in Egypt and is closely associated with decreased pulmonary function. Improving nutritional status through early intervention, routine screening, and comprehensive care can significantly enhance lung function, growth, and overall quality of life in this vulnerable population. Addressing the nutritional needs of patients with CF should be a priority for healthcare providers, policymakers, and caregivers alike to improve both short-term outcomes and long-term prognosis.

## Figures and Tables

**Table 1:** Patients’ clinical characteristics.

Variable Patients with CF		
Current age (years)	Range	3–18
	Mean (SD)	7.5 (3.3)
Age at diagnosis (months)	Range	0.5–96
	Mean (SD)	18.42 (7.6)
Sex (N: %)	Male	54 (52%)
	Female	50 (48%)
Sweat chloride test (mmol/L)	Minimum–Maximum	61–179
	Mean (SD)	99 (36)
Consanguinity		42 (40.4%)
Family history of CF		8 (7.6%)
Pulmonary manifestations		104 (100%)
Chronic cough		100 (96%)
Sinusitis		23 (22%)
Clubbing		19 (18.3%)
Bronchiectasis		11 (10.5%)
Hemoptysis		4 (3.8%)
Gastrointestinal manifestations		22 (21%)
Steatorrhea		22 (21%)
Hepatomegaly		4 (3.8%)
Cholelithiasis		5 (4.8%)
Diarrhea		4 (3.8%)
Cystic fibrosis-related diabetes		5 (4.8%)
Pancreatic insufficiency		88 (84.6%)
Gene analysis:	Class I	6 (5.8%)
	Class II	19 (18.3%)
	Class III	1 (0.96%)
	Class IV	2 (1.93%)
	No data	76 (73.1%)
STAMP nutritional risk screening:		
High risk (N: %)		76 (73.1%)
Moderate risk (N: %)		28 (26.9%)
Nutritional status (BMI Z-scores): [N: %]		
Mild malnutrition		25 (24%)
Moderate malnutrition		14 (13.5%)
Severe malnutrition		36 (34.6%)
Normal		29 (27.9%)

STAMP: Screening Tool for the Assessment of Malnutrition in Pediatrics.

**Table 2:** Comparison between the malnutrition and the normal nutrition groups.

Variable	Nutritional Status				*p*-Values		
	Mild Malnutrition (*n* = 25)	Moderate Malnutrition (*n* = 14)	Severe Malnutrition (*n* = 36)	Normal Nutrition Group (*n* = 29)	P1 Value	P2 Value	P3 Value
Male [number (%)]	12 (48%)	7 (50%)	19 (52.7%)	16 (55%)	NS	NS	NS
Age [mean (SD); year]	6.8 (2.3)	7.8 (1.9)	6.9 (2.2)	7.4 (2.5)	NS	NS	NS
Body weight (kg); median	19.9	14.8	12.2	27	<0.05 *	<0.001 *	<0.001 *
Number of hospitalizations [median; times]	4.77 (2.00, 6.00)	5.04 (2.00, 6.00)	5.08 (2.00, 6.00)	4.76 (1.00, 6.00)	NS	NS	NS
Total protein [mean (SD); g/dL]	7.3 (1.1)	7.2 (1.2)	6.8 (2.2)	7.8 (1.9)	NS	NS	NS
Albumin [median; g/dL]	3.68 (3.03, 4.98)	3.72 (3.00, 4.17)	3.30 (3.06, 3.88)	4.63 (3.9, 4.68)	<0.001 *	<0.001 *	<0.001 *
Hemoglobin [mean (SD); year]	12.6 (1.9)	12.1 (1.7)	11.7 (1.12)	12.9 (1.5)	NS	NS	NS
Sweat chloride concentration [mean (SD) mmol/L]	99 (29)	98 (36)	101 (28)	103 (24)	NS	NS	NS

* Significant; NS = non-significant. P1 between the mild malnutrition and the normal group, P2 between the moderate malnutrition and the normal group, and P3 between the severe malnutrition and the normal group.

**Table 3:** Comparison of lung function between malnutrition and normal nutrition groups.

Variable	Nutritional Status				*p*-Values		
	Mild Malnutrition (*n* = 18)	Moderate Malnutrition (*n* = 12)	Severe Malnutrition (*n* = 25)	Normal Nutrition Group (*n* = 24)	P1 Value	P2 Value	P3 Value
VC	1.97 (0.69)	1.87 (0.76)	1.79 (0.74)	2.35 (0.84)	<0.03 *	<0.001 *	<0.0001 *
FVC (% pred)	84 (10)	82 (14)	79 (12)	91 (11)	0.01 *	0.01 *	<0.0001 *
FEV1	1.72 (0.52)	1.66 (0.73)	1.58 (0.68)	2.21 (0.49)	<0.0001 *	<0.0001 *	<0.0001 *
FEV1 (% pred)	78 (18)	72 (20)	72 (15)	92 (19)	<0.001 *	<0.001 *	<0.001 *
FEV1/FVC ratio (%)	86.9 (10)	88.1 (8)	88.2 (9)	94 (6)	0.001 *	0.001 *	0.001 *
PEF (% pred)	69 (11)	62 (14)	60 (12)	82 (18)	0.01 *	0.001 *	0.001 *
FEF 25 (% pred)	68 (11)	63 (16)	61 (10)	80 (17)	0.001 *	0.001 *	0.001 *
FEF 50 (% pred)	52 (17)	50 (20)	51 (16)	77 (21)	0.001 *	0.001 *	0.001 *
FEF 75 (% pred)	33 (10)	29 (13)	30 (14)	53 (16)	0.001 *	0.001 *	0.001 *

VC: vital capacity; FVC: forced vital capacity; % pred: the percentage of the predicted value; FEV1: forced expiratory volume in one second; PEF: peak expiratory flow; and FEF: forced expiratory flow at 25%, 50%, and 75% of forced vital capacity as a percentage of the predicted value. * Significant *p*-value (<0.05). P1 = (mild vs. normal), P2 = (moderate vs. normal), and P3 = (severe vs. normal).

**Table 4:** Correlation coefficient between BMI Z-scores and pulmonary function parameters.

Pulmonary Function Parameter	Correlation Coefficient (r)	*p*-Value
FVC (% pred)	0.47	0.001 *
FEV1 (% pred)	0.54	0.002 *
FEV1/FVC ratio (%)	0.76	<0.001 *
PEF (% pred)	0.67	<0.001 *
FEF 25 (% pred)	0.5	0.001 *
FEF 50 (% pred)	0.54	0.002 *
FEF 75 (% pred)	0.56	0.001 *

VC: vital capacity; FVC: forced vital capacity; % pred: the percentage of the predicted value; FEV1: forced expiratory volume in one second; PEF: peak expiratory flow; and FEF: forced expiratory flow at 25%, 50%, and 75% of forced vital capacity as a percentage of the predicted value. * Significant.

## Data Availability

The datasets generated and analyzed during the current study are available from the corresponding author upon reasonable request.
